# Modulating the Geometry of the Carbon Nanofiber Electrodes Provides Control over Dopamine Sensor Performance

**DOI:** 10.1021/acs.analchem.2c04843

**Published:** 2023-01-26

**Authors:** Ayesha Kousar, Ishan Pande, Laura F. Pascual, Emilia Peltola, Jani Sainio, Tomi Laurila

**Affiliations:** † Department of Electrical Engineering and Automation, School of Electrical Engineering, Aalto University, P.O. Box 13500, 00076 Aalto, Finland; ‡ Department of Mechanical and Materials Engineering, Faculty of Technology, University of Turku, Vesilinnantie 5, 20500 Turku, Finland; § Department of Applied Physics, School of Science, Aalto University, P.O. Box 15100, 00076 Aalto, Finland; ∥ Department of Chemistry and Materials Science, School of Chemical Engineering, Aalto University, P.O. Box 16200, 00076 Aalto, Finland

## Abstract

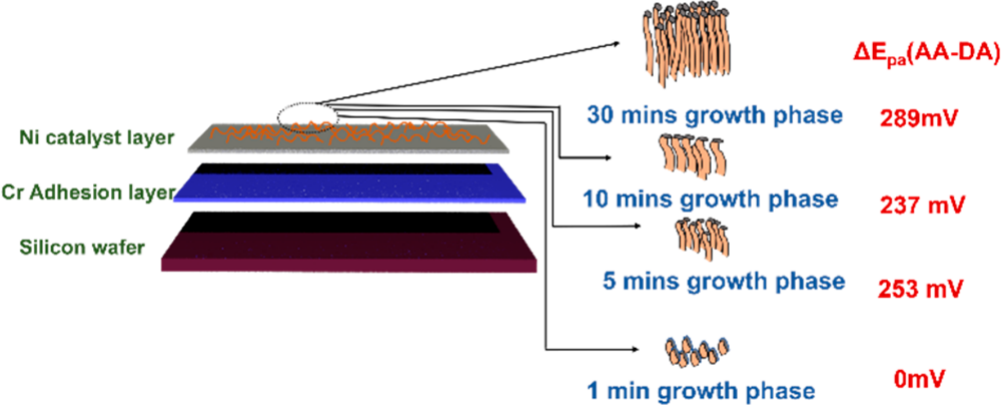

One of the major challenges for in vivo electrochemical measurements of dopamine (DA) is to achieve selectivity in the presence of interferents, such as ascorbic acid (AA) and uric acid (UA). Complicated multimaterial structures and ill-defined pretreatments have been frequently utilized to enhance selectivity. The lack of control over the realized structures has prevented establishing associations between the achieved selectivity and the electrode structure. Owing to their easily tailorable structure, carbon nanofiber (CNF) electrodes have become promising materials for neurobiological applications. Here, a novel yet simple strategy to control the sensitivity and selectivity of CNF electrodes toward DA is reported. It consists of adjusting the lengths of CNF by modulating the growth phase during the fabrication process while keeping the surface chemistries similar. It was observed that the sensitivity of the CNF electrodes toward DA was enhanced with the increase in the fiber lengths. More importantly, the increase in the fiber length induced (i) *an anodic shift* in the DA oxidation peak and (ii) *a cathodic shift* in the AA oxidation peak. As the UA oxidation peak remained unaffected at high anodic potentials, the electrodes with long CNFs showed excellent selectivity. Electrodes without proper fibers showed only a single broad peak in the solution of AA, DA, and UA, completely lacking the ability to discriminate DA. Hence, the simple strategy of controlling CNF length without the need to carry out any complex chemical treatments provides us a feasible and robust route to fabricate electrode materials for neurotransmitter detection with excellent sensitivity and selectivity.

## Introduction

The progress in the engineering of carbon nanomaterials is revolutionizing the various aspects of the scientific world whether it be energy storage, bioimaging, wearable electronics, solar cells, drug delivery, or biosensing.^1−5^ Plenty of research has been performed to study the properties of different forms of carbon nanomaterials such as carbon nanotubes, carbon nanofibers, carbon nanospikes, and carbon nanotube yarns as electrode materials for the neurobiological measurements.^6−9^ These forms of carbon nanomaterials have been proved to be excellent materials for sensing neurochemicals by possessing one or other of the properties such as high sensitivity, wide potential window, good stability, and reversibility. However, in comparison to other forms, carbon nanofibers stand out as a superior sensor material due to their ability to retain the vertically aligned geometry with the fiber lengths reaching from tens of nanometers to tens of micrometers, better isolation among the nanostructures, improved signal-to-noise ratio due to the presence of underlying metal layers, and decreased capacitance.^10,11^ By fine-tuning the fabrication parameters, the microstructure of CNFs can be controlled, providing the immense potential of modulating their properties selectively depending upon the target application. The literature is full of studies comparing the sensor performance of different carbon nanomaterial electrodes with varying geometries (or dimensions) associated with nonidentical chemistries.^12^ This makes rationalization of the results and extracting any kind of structure–property relationships from them extremely difficult. Modulating the geometrical features and aspect ratios of nanostructures of a single type of carbon nanomaterial with similar chemistry and employing these features to tune the performance of DA sensors have not been studied to the best of our knowledge.

Selective detection of DA and its coexisting neurochemicals such as AA and UA has been a critical, widely debated, and an old question in neuroelectrochemistry.^13,14^ Various research papers have been published on selective detection of dopamine by using negatively charged membranes or coatings of materials such as Nafion, polypyrrole, laccase, and polyaniline by utilizing their cationic permeability.^15−18^ Similarly, pretreatment and various modifications of electrodes have been performed to achieve selective dopamine sensing.^19,20^ Differential pulse voltammetry has also been utilized as a means to show potential shifts, thus maximizing the peak separation among DA, UA, and AA.^21,22^ However, there are challenges associated with these methods such as slow measurements with DPV, slow response time, reproducibility and stability of the electrode coatings, ill-defined fabrication procedures, and lack of control over the modified electrodes. In this work, we propose to replace these complex and challenging modification methods with a simple but accurate control of CNF lengths.

The goal of this study is to evaluate and rationalize the effects of CNF length on DA sensor performance and present a model system to demonstrate the DA sensor selectivity and sensitivity. We will show that by (i) tailoring the CNF growth parameters, it is possible to (ii) control the DA sensor properties and (iii) increase both the sensitivity and selectivity. To show this, CNF electrodes with different lengths were fabricated by varying the duration of the growth phase to 1, 5, 10, and 30 min. The silicon substrates were coated with 80 nm of Cr as an adhesive layer, and 20 nm of Ni was used as a catalyst to grow CNF for all cases studied here.^23^ The fabrication conditions were identical for all the electrodes except the growth duration, thus providing us the CNF with different lengths yet similar surface chemistries. Electrochemistry of DA, AA, and UA was studied on CNFs with different lengths, and redox kinetics of all the analytes (inner sphere reaction, ISR) were assessed. The experiments unambiguously show that the CNF length plays an important role in dictating the sensitivity and selectivity of DA sensors and point toward the potential of controlling and fine-tuning the sensor performance simply by adjusting CNF length.

## Materials and Methods

### Fabrication of CNF

All samples were processed on p-type Si wafers (Sigert Wafers, Germany) as a substrate material. Si wafers were coated with the metal adhesion and catalyst layer as a first step of the process. Cr metal of 80 nm thickness was deposited as an adhesion layer followed by 20 nm of Ni as a catalyst/seed layer deposition. Metal layers were deposited using an electron beam evaporator (MASA IM-9912). The chamber pressure was maintained around 2 × 10^–7^. After the coating, dicing of the substrate was performed, and 7 mm × 7 mm pieces were prepared. A PECVD reactor (Aixtron black magic) was used for growing the CNF on top of the metal-coated silicon substrates. The CNF growth process in PECVD was carried out in a series of steps. As a first step, the chamber pressure was taken down to 0.1 mbar followed by heating the chamber to 400 °C with a ramp speed of 250 °C/min. 100 sccm NH_3_ buffer was introduced into the chamber when the temperature reached 395 °C. Subsequently, the chamber was heated to 600 °C with a ramp rate of 300 °C/min. A 230 W DC plasma was ignited at 575 °C. Simultaneously, the NH_3_ flow was increased to 125 sccm, and 30 sccm C_2_H_2_ was injected into the chamber. Different lengths of CNF were obtained by maintaining these parameters for 1, 5, 10, and 30 min. The chamber pressure was recorded to be 3 mbar during the fabrication process.

### Electrode Fabrication

CNF electrodes were done by placing a piece of sample on top of a conductive copper clap (double sided FR 4 glass fiber substrate with thickness of 0.3 mm). The sample was enclosed with inert PTFE-tape (Saint-Gobain Performance Plastics CHR 2255-2) with a 3 mm hole (radius = 1.5 mm), which was placed on top of the carbon sample to define the working area of the electrode and isolate the copper from the electrolyte. The contact between substrate and carbon sample was enhanced by scraping the back side of the carbon sample with a piece of copper.

### Scanning Electron Microscopy

The morphology of CNF samples was studied using a scanning electron microscope (SEM) (Zeiss Supra 40 and Zeiss Sigma VP). The length and diameter analysis was carried out in ImageJ. Lengths and diameters of 20–30 CNF were measured, and the average and the standard deviation are provided.

### Electrochemical Measurements

Electrochemical measurements were carried out using both conventional cyclic voltammetry (CV) and using the rotating disk electrode (RDE) configuration. A Gamry Reference potentiostat was used with a three-electrode setup containing an Ag/AgCl reference electrode and a platinum wire as a counter electrode for the electrochemical measurements. The solutions were purged with N_2_ gas for 10 min before the measurements. Newly prepared electrodes (*r* = 3 mm) were used for each electrochemical measurement. The uncompensated resistance (Ru) was measured for each electrode in PBS. The PBS solution was prepared by mixing 8 g of NaCl, 1.44 g of NaHPO_4_, 0.2 g of KCl, and 0.24 g of KH_2_PO_4_ in 1 L of distilled water. The pH of the solution was maintained at 7.4 using 2 M NaOH or HCl. Dopamine hydrochloride (Sigma-Aldrich) in the presence of AA (Merck) and UA (Sigma) was used to evaluate the selective detection ability of the electrodes in the PBS solution. Rotational frequencies of ω = 300, 1500, 2700, 3900, and 5000 rpm were used to conduct RDE measurements. All the measurements were carried out at room temperature in the Faraday cage. The average values and standard deviation of 3–4 samples are provided.

### X-ray Photoelectron Spectroscopy (XPS) Measurements

X-ray photoelectron spectroscopy (XPS) was carried out with a Kratos Axis Ultra spectrometer with monochromated Al Kα radiation, a pass energy of 40 eV, an X-ray power of 75 W, and an analysis area of approximately 700 μm × 300 μm. The binding energy scale was based on instrument calibration, and no additional binding energy correction was applied to the data. The elemental composition was determined from peak areas of high-resolution core level spectra after Shirley background subtraction using equipment specific sensitivity factors. Peak fitting was done using Gaussian–Lorentzian peaks (GL (30) line shape in CasaXPS) with the positions of the peaks fixed to within ±0.1 eV of given binding energies. For sp^2^ carbon an asymmetric line shape was used in CasaXPS.^24^ The full widths at half-maximum (FWHMs) of the peaks were restricted to be equal within a fit with the exception of the sp^2^ carbon peak.

### Raman Spectroscopy

Raman spectroscopy was performed with micro-Raman spectroscope (WITec Alpha RA+) equipped with an optical microscope. Objective lens of 50× and laser with excitation wavelength of 532 nm were used. Single spectrum with 10 accumulations was captured using integration time of 0.5 s.

## Results and Discussion

### Structural Characterization

**Figure 1:**
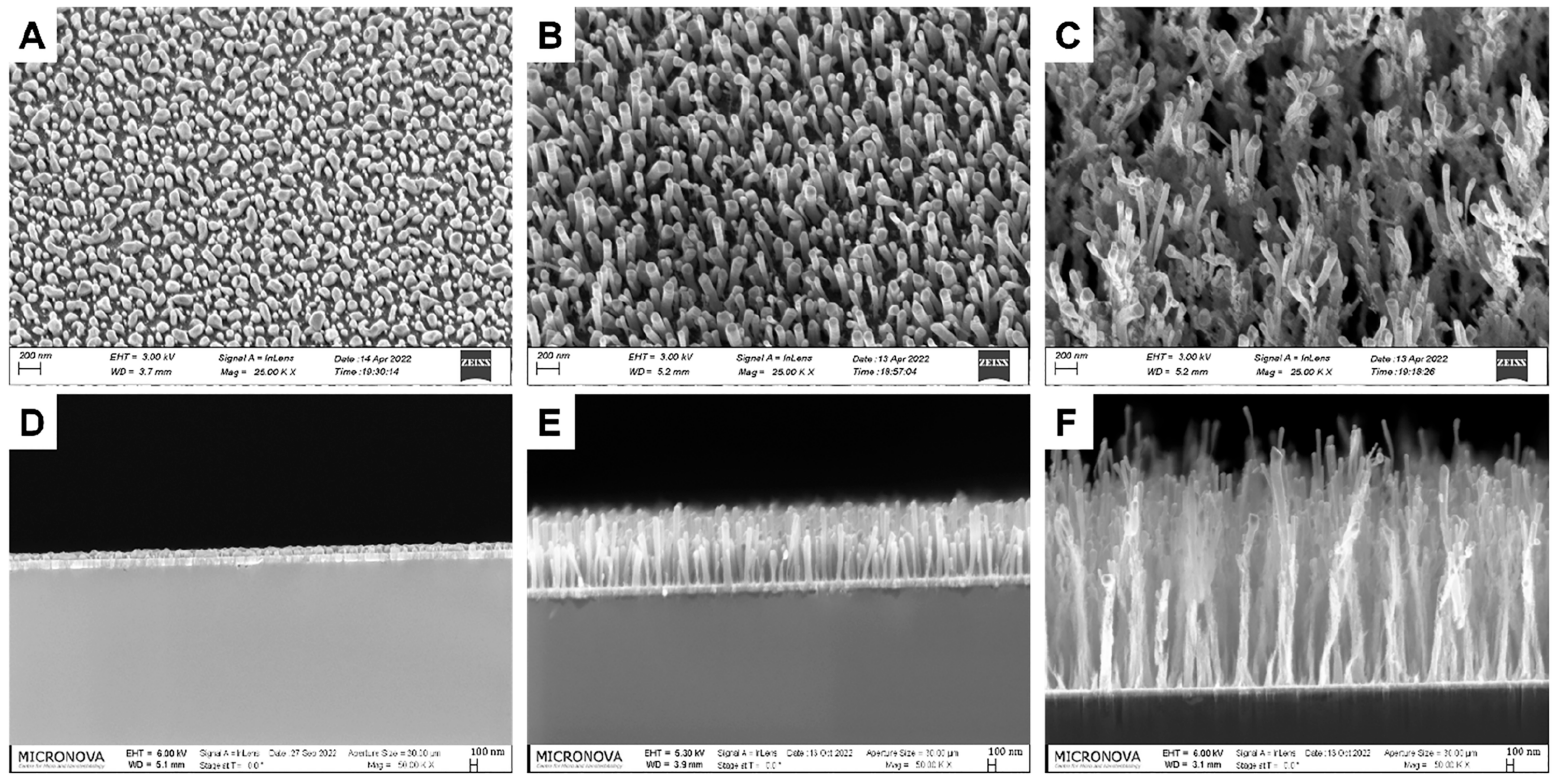
SEM images of CNF electrodes captured from the top by tilting the sample stage to 25° and of cross sections for (A, D) CNF-1min, (B, E) CNF-5mins, and (C, F) CNF-30mins.

Overall morphology and the macrostructure of the CNFs were investigated by SEM micrographs (Figures 1 and S1). CNF grown for different durations showed differences in the overall morphology. The increase in the lengths of CNF as a function of the growth phase is apparent (Table 1). Also, it is evident from the SEM micrographs that the CNF-1min showed primarily the metal nanoparticles island formation with not yet visible growth of CNFs. The variation in diameter is a bit higher in the CNF-10mins where fibers with slightly larger diameter and lower population density can be observed in comparison to CNF-5mins. Some fibers were observed to bunch together, making clumps and clusters, especially after 30 min growth, yet mainly maintaining the vertically aligned orientation.

**Table 1:** Physical–Chemical Properties of CNFs with Different Dimensions and Electrochemistry of [Ru(NH_3_)_6_]^3+^ in 1 M KCl on These Electrodes at 100 mV/s *v.*

sample	length (nm)	diameter (nm)	pseudocapacitance (μF/cm^2^)	potential window (PBS)	relative surface area^a^	Δ*E*_p_ (mV)	*I*_pa_ (μA)	*I*_pa_/*I*_pc_	*k*_0_ (cm/s)
CNF-30mins	2684 ± 436	64 ± 15	773 ± 112	1.16 ± 0.03	∼25×	59 ± 3	13 ± 1	0.86 ± 0.03	0.214 ± 0.056
CNF-10mins	789 ± 117	96 ± 22	243 ± 69	1.32 ± 0.07	∼6×	61 ± 3	12 ± 1	0.90 ± 0.04	0.157 ± 0.070
CNF-5mins	600 ± 132	72 ± 15	312 ± 44	1.37 ± 0.02	∼6×	61 ± 2	11 ± 0	0.92 ± 0.05	0.153 ± 0.046
CNF-1min	100 ± 18	92 ± 19	85 ± 12	1.46 ± 0.01	1×	62 ± 6	12 ± 1	0.91 ± 0.01	0.140 ± 0.093

a With respect to CNF-1min.

### Surface Chemistry

**Figure 2:**
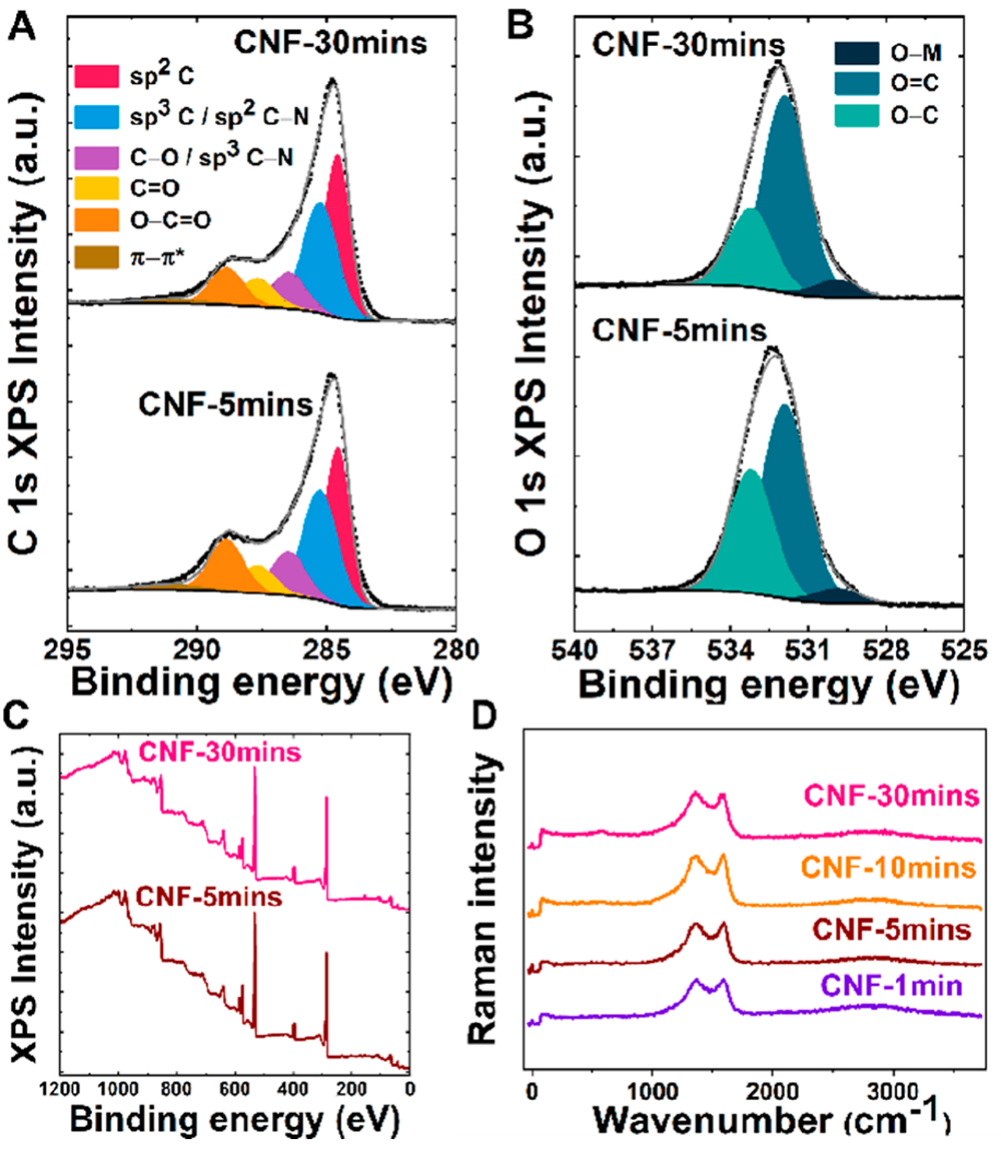
X-ray photoelectron spectra of CNF-5mins and CNF-30mins: (A) C 1s region; (B) O 1s region. (C) Survey spectra and (D) Raman spectra of CNF.

XPS was used to study the chemical composition of the CNF-5mins and CNF-30mins. These two lengths were studied to generalize the surface chemistry changes in CNF electrodes as the fibers elongate. Figure 2A–C includes the carbon 1s, oxygen 1s, and survey spectra. Atomic concentrations are provided in Table S2. Figure 2A shows the C 1s regions for both CNF electrodes. As can be seen from the figure, the C 1s spectra for both CNF samples are similar. The spectra have been fitted with six components: sp^2^ carbon (284.5 eV); sp^3^ carbon (285.2 eV), which could include contributions from sp^2^ C–N bonds; C–O–C and/or C–OH (286.4 eV), which could include contributions from sp^3^ C–N bonds; C=O (287.6 eV), O–C=O (288.8 eV), and a π–π* shake up transition (291.0 eV).^25,26^ However, because of the complexity and high number of possible components, this peak fitting should be considered somewhat tentative. As shown in Figure 2B, a rather broad O 1s peak has been fitted with three components corresponding to metal–oxygen (O-M) bonds (529.9 eV), O=C bonds (531.9 eV), and O–C and/or OH–C bonds (533.2 eV).^25,27^ The peak for metal–oxygen bonds could be related to oxidized nickel and/or chromium. The concentrations of the different components derived from peak fitting are given in Table S2, which confirm that only small differences in the relative amounts of chemical species are present. The survey spectra for CNF-5mins and CNF-30mins shown in Figure 2C confirm that the chemical composition is similar, and no additional unexpected elements are found.

Additionally, Raman spectroscopy was performed to evaluate possible changes in the surface chemistry as a result of increase in the CNF lengths (Figure 2D). Raman spectra of the CNF with varying lengths showed the D and G peaks and 2D peak at the same position (1358 and 1589 and 2850 cm^–1^, respectively) indicating that the proportion of sp^3^ content and graphitic carbon is similar despite the changes in the length.^28^ Moreover, the *I*_D_/*I*_G_ value is approximately 1 in all CNF lengths pointing toward the presence of large number of defects and suggesting that the increase in trunk of the fibers is not causing significant changes in the surface chemistry or fiber microstructure.

### Electrochemical Properties

Pseudocapacitance (*C*_dl_) of the CNF electrodes was highest for CNF-30mins while showing a decrease with the decrease in the lengths of fibers (Table 1). There are no significant differences between *C*_dl_ for the CNF-10mins and CNF-5mins, indicating that the fiber lengths were not significantly different consistent with the SEM results. The rough surface area estimation based on the measured *C*_dl_ shows that the CNF-30mins have approximately 4-fold the surface area of 5–10 min grown samples and approximately 25 times the surface area of CNF-1min. The potential window of the CNF electrodes also became narrower with the increase in the CNF lengths (Table 1).^23^

The results showing the effect of different lengths of CNF on the apparent electron transfer kinetics estimated through cyclic voltammetry measurements with OSR probe ([Ru(NH_3_)_6_]^3+^) have been described in detail in ref (23). Briefly, changes in the CNF dimensions did not induce substantial differences in oxidation peak current (*I*_pa_) in 1 mM [Ru(NH_3_)_6_]^3+^ at both 100 and 400 mV/s scan rates (Tables 1 and S1). This indicates that the electrochemically active area for OSR reaction is in a similar range despite the increase in the apparent surface area based on the pseudocapacitance measurements. It is to be noted that electrochemically active surface area (ECAS) is highly probe dependent, and thus similar values for different probes should not be expected. Therefore, we do not provide any numerical values for ECAS in the following discussion but instead compare internally the trends among the CNF data set using various redox probes. Peak separation (Δ*E*_p_) values at 100 mV/s and 400 mV/s are provided as a measure to study the reaction kinetics, as well as the effect of any thin layer formation.^23^ Differences in the CNF lengths did not cause noticeable differences in Δ*E*_p_, and the electron transfer kinetics were nearly reversible at 100 mV/s. *I*_pa_/*I*_pc_ values did not differ noticeably with the changes in the lengths of the fibers and confirmed the electron transfer to be nearly reversible in all cases. However, a significant drop in Δ*E*_p_ for CNF-30mins at 400 mV/was seen (Table S1). The reduction of Δ*E*_p_ and an apparent increase in *k*_0_ at 400 mV/s for CNF-30mins can be explained by the matching of CNF lengths with the diffusion layer thickness that results in the thin liquid layer formation.^23^ The main implication for this study is that we need to consider the possible thin liquid layer effects while analyzing the electrochemical results for different analytes.

### DA Sensitivity

**Figure 3:**
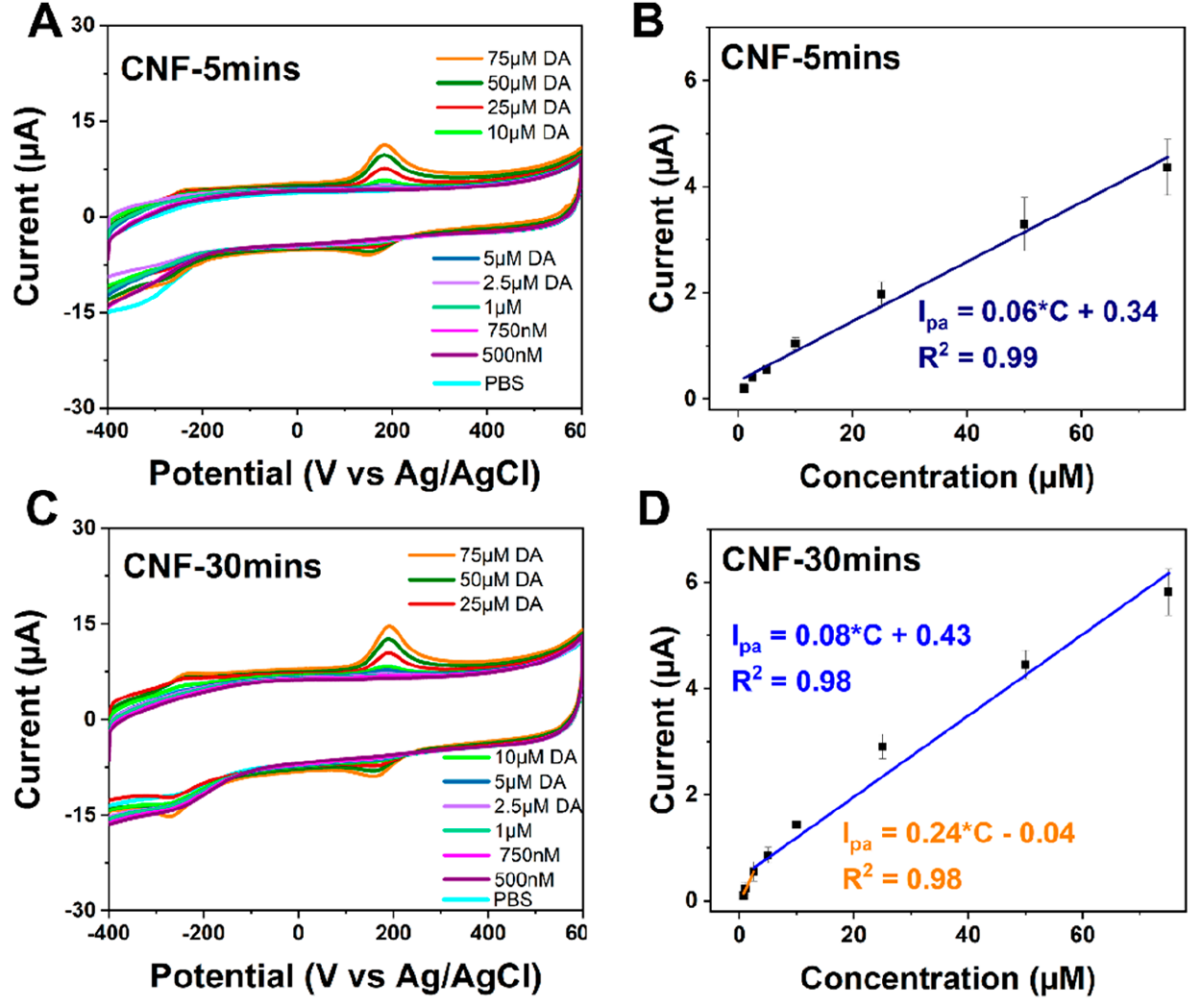
CV response of (A) CNF-5mins and (C) CNF-30mins in PBS electrolyte containing different concentrations of DA. Linear response of oxidation current as a function of concentration for (B) CNF-5mins and (D) CNF-30mins electrodes.

The DA sensitivity measurements were performed by recording CVs at several concentrations of DA in PBS (Figures 3 and S2). It is evident from the Figure 3 that the shape of the CVs becomes more symmetric with the increase in the length of the fibers. The diffusion tail being observed in CNF-1min electrodes nearly disappears in the case of CNF-30mins. The CVs showed a large anodic peak for all electrodes depicting DA oxidation to dopamine-*o*-quinone (DOQ) by a two-electron transfer process. The oxidation peak position did not show distinct differences with different fiber lengths as the average value of the oxidation potential (*E*_pa_) was in the range of 179–182 mV. However, the onset potential for CNF-30mins was slightly higher in comparison to those of shorter fibers. This indicates that the activation energy for DA reaction on CNF-30mins electrodes is higher in comparison to the CNF with shorter fibers, which can be caused by the stronger interaction of DA with the electrode surface as the lengths of fibers increase. The reaction kinetics were nearly reversible for CNF-30mins, CNF-10mins, and CNF-5mins, and no significant differences were observed in Δ*E*_p_ values at 100 mV/s (Table 2). However, Δ*E*_p_ was significantly higher for the CNF-1min electrode indicating slower kinetics. This implies that the reaction kinetics of DA become fast with the increase in the length of the fibers up to a certain limit and become unaffected afterward. Note that in the absence of any CNFs (only Cr/Ni layers) the electrodes do not detect DA at all (Figure S3), thus confirming that the CNFs, even very short ones, are needed to achieve the successful measurement.

**Table 2:** Electrochemistry of DA on the Electrodes with Different Fiber Lengths Determined from CV Measurements

					slope of log *I*_pa_ vs log *v*				
electrode	*I*_pa_ (μA)	onset potential (mV)	*E*_pa_ (mV)	Δ*E*_p_ (mV)	(100 μM)	(5 μM)	linear range (μM)	sensitivity (A M^–1^ cm^–2^)	LOD (nM)
CNF-30mins	5 ± 1	135 ± 1	182 ± 4	33 ± 3	0.70 ± 0.02	0.92 ± 0.09	0.75–75	1.090	319
CNF-10mins	4 ± 0	123 ± 4	182 ± 9	34 ± 1	0.61 ± 0.05	0.85 ± 0.05	0.75–75	0.079	221
CNF-5mins	4 ± 1	125 ± 2	179 ± 1	31 ± 1	0.62 ± 0.04	0.80 ± 0.03	1–75	0.080	254
CNF-1min	3 ± 0	123 ± 3	182 ± 3	42 ± 4	0.53 ± 0.00	0.73 ± 0.04	0.75–75	0.056	750

The oxidation current vs concentration plots in the range of 0.5–75 μM DA at 100 mV/s as well as respective CVs at different DA concentrations are given in Figures 3 and S2. The slope of oxidation current vs DA concentration increased with the increase in the length of fibers (Table 2). These results imply that the sensitivity of the electrodes improves for the longer CNF electrodes. Due to the variations in the standard deviations in blank PBS for these samples, there is a slight variation in the limit-of-detection (LOD) trend in comparison to sensitivity results. Furthermore, it is evident that the electrodes with longer fibers showed two linear regions showing higher slopes at the lower concentrations while slopes became less steep at higher concentrations of DA (Figure 3D). This implies that the contribution of adsorption of DA is stronger on the electrode surface at low DA concentrations in comparison to the higher concentrations.^29^

The *I*_pa_ values of DA at 100 μM DA (with 100 mV/s) were observed to be highest for the longest CNF samples indicating the highest ECAS for DA (Table 2). CNF-10mins and CNF-5mins showed *I*_pa_ of approximately 4 μA, indicating the similar electrochemically active area. The CNF-1min showed *I*_pa_ of 3 ± 0 μA, demonstrating the smallest electrochemically active area. The enhancement in the peak intensities suggests that the increase in the length of the trunks of the fibers provides more adsorption sites for DA resulting in enhanced oxidation currents. Slopes of log *I*_pa_ vs log *v* plots were calculated to predict the effect of lengths of CNF on the contribution of diffusion and adsorption toward the total current at 5 μM and 100 μM DA concentrations. At 100 μM DA concentration, there is a continuous increase in the slope values as a function of fiber length (Table 2). These results indicate that the importance of adsorption increases as a function of increasing lengths of the fibers. The slopes at lower concentration of 5 μM DA were substantially higher (≈0.73–0.92) as expected and showed an increasing trend with the increase in the lengths. The results are consistent with the ones presented above, showing the enhanced adsorption as the fibers elongate and at lower DA concentrations.

As thin liquid layer formation is possible in this system under a specific scan rate range,^23^ we investigated the Δ*E*_p_ changes occurring as a function of scan rate (0.01–2.5 V/s) to differentiate between the adsorption and thin layer formation effects for DA reaction at 100 μM DA concentration (Figure S4). Respective CVs show that the peak separation is monotonically increasing with the increase in scan rate, indicating sluggish kinetics (Figures S4 and S5). Thus, we did not see the lowering of the peak separation versus scan rate, which is considered a typical parameter to spot the thin layer formation.^23^ Hence, we can rule out any noticeable thin liquid layer formation contribution to the DA reaction.

**Figure 4:**
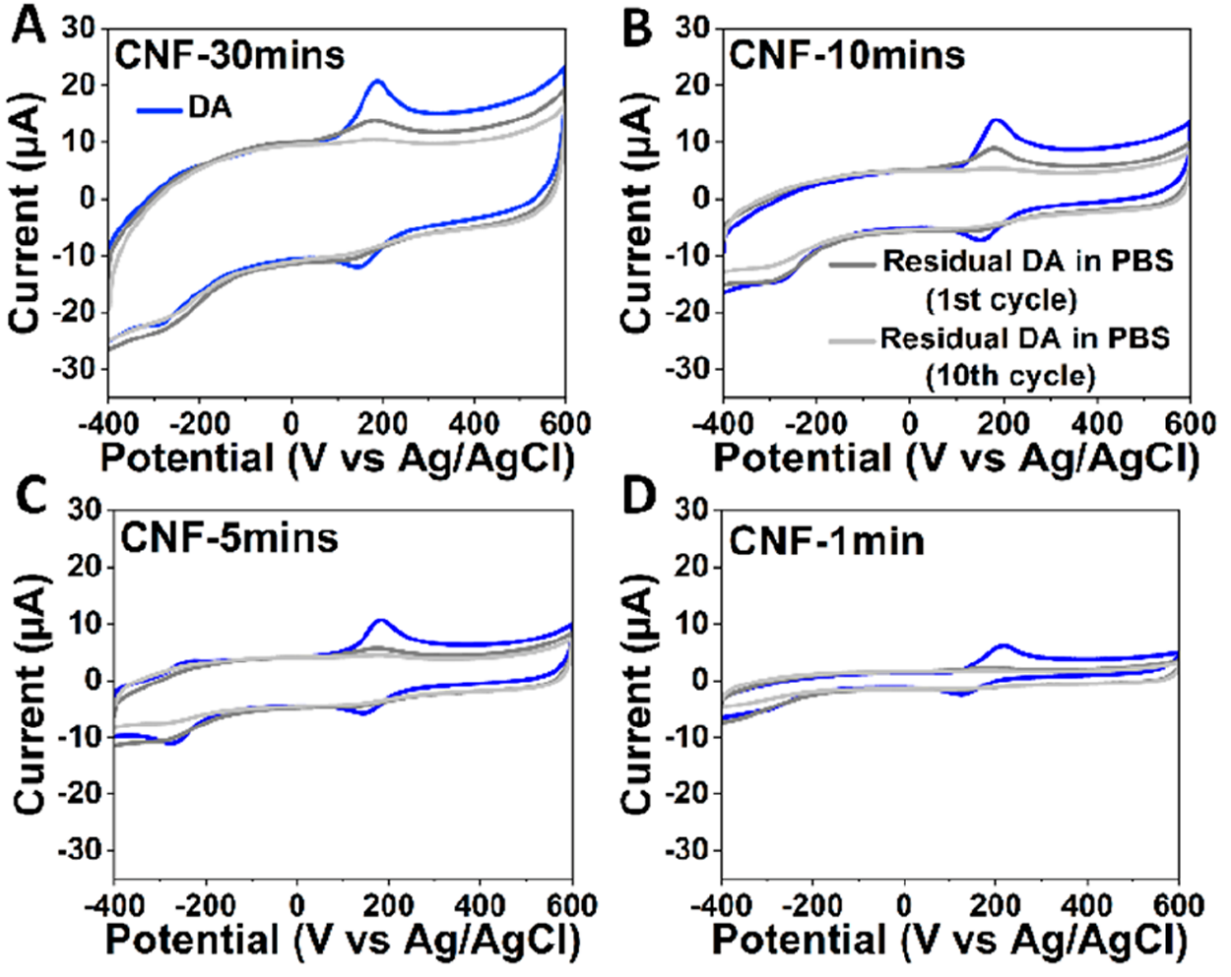
Cyclic voltammograms for (A) CNF-30mins, (B) CNF-10mins, (C) CNF-5mins, and (D) CNF-1min in 100 μM DA (shown in blue) and during washing in blank PBS (dark gray, first cycle; light gray, 10th cycle) (*v* = 100 mV/s).

To further rule out the thin layer formation effects in DA electrochemistry on these electrodes and confirm the role of adsorption, electrodes were washed in PBS after DA detection, and their behavior in blank PBS was recorded (Figure 4). All electrodes were subjected to 30 cycles of scanning at 100 mV/s in 100 μM DA solution followed by washing in blank PBS for 20 cycles. The amount of residual DA adhered to the electrode surface in the CV obtained in the blank PBS was used to estimate the adsorption contribution. Interestingly, small DA peaks were observed on the electrodes even after washing for 10 cycles. The residual peak current was highest for CNF-30mins and showed a decreasing trend with the decrease in the fiber lengths. These results confirm that the contribution of adsorption is increasing with the increase in the fiber length. Results are also consistent with the observed anodic shift of the DA oxidation potential with CNF-30mins electrodes.

### AA Electrochemistry

**Figure 5:**
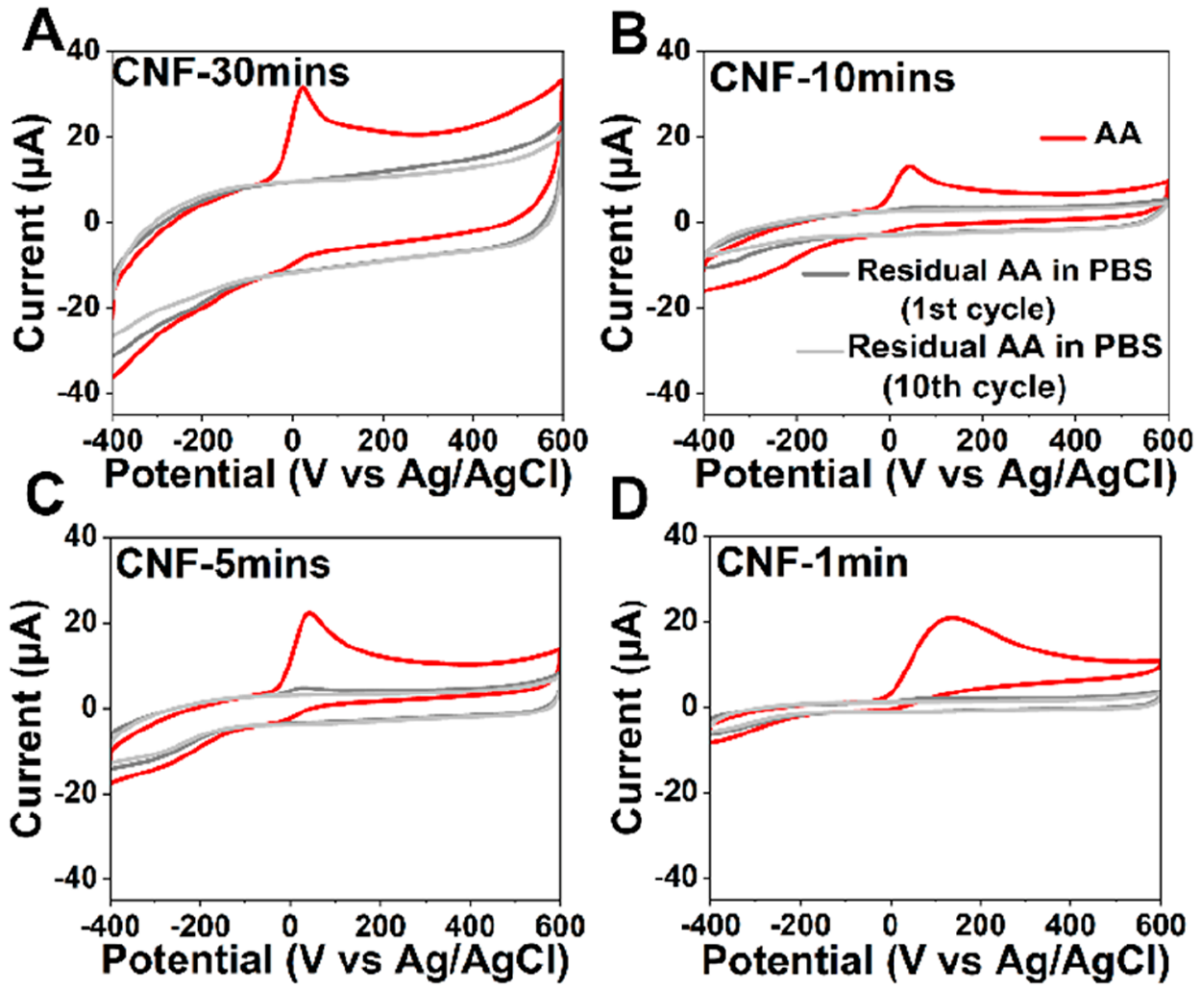
CV response of (A) CNF-30mins, (B) CNF-10mins, (C) CNF-5mins, and (D) CNF-1min in 1 mM ascorbic acid (shown in red) and during washing in blank PBS at 100 mV/s (dark gray, first cycle; light gray, 10th cycle).

The effect of changing the dimensions of CNF on the AA electrochemistry is shown in CVs recorded in 1 mM AA in PBS (Figure 5). A broad anodic peak assigned to AA oxidation to dehydroascorbic acid is observed in the case of the CNF-1min electrode; however, it becomes narrower and sharp as the fibers elongate. The *I*_pa_ values for AA slightly increase with the increase in the length of fibers (Table 3). These results indicate that the ECAS for AA redox reaction shows a slight increase as the fibers elongate from a few hundred nanometers to a few micrometers. For CNF-1min electrodes, *I*_pa_ and hence the ECAS for this reaction are roughly halved in comparison to the CNF-30mins. Interestingly, the oxidation peak of AA shifts to a cathodic direction as the fibers lengthen. The longest CNF electrodes showed AA oxidation at 21 ± 2 mV, fibers with 10 and 5 min growth durations show AA oxidation at 42 ± 9 and 43 ± 8 mV, while the CNF-1min electrodes undergo oxidation at 114 ± 1 mV. The cathodic shift of the AA peak for longer fibers indicates that (i) the dehydroascorbic acid is adsorbing on the CNFs and (ii) the effect is stronger as the fibers become longer.

**Table 3:** Electrochemistry of Ascorbic Acid on CNF Electrodes with Different Lengths

	1 mM AA		
electrode	onset potential (mV)	*E*_pa_ (mV)	*I*_pa_ (μA)
CNF-30mins	–33 ± 1	21 ± 2	17 ± 1
CNF-10mins	–23 ± 4	42 ± 9	15 ± 2
CNF-5mins	–23 ± 3	43 ± 8	12 ± 0
CNF-1min	16 ± 3	114 ± 1	9 ± 1

To confirm that the shifts are caused by adsorption and not by thin liquid layer formation also in this case, we carried out the washout experiments in blank PBS (Figure 5). The electrodes were cycled in 1 mM AA for 30 cycles in PBS followed by washing in the blank PBS for 20 cycles at 100 mV/s. We did not see any noticeable residual AA attached to the surface during washing as expected. There was a minor accumulation of AA seen on 5 min and 10 min electrodes during the first cycle of washout, which disappeared quickly during the following cycles, and CV observed at the 10th cycle did not show any evidence of adherence of AA (unlike the case with DA). During the electrochemical reaction, AA oxidizes into dehydroascorbic acid, which then undergoes the irreversible hydration reaction forming electrochemically inactive 2, 3-diketogulonic acid.^13^ Thus, the absence of any residual AA on electrode indicates that the oxidation reaction to dehydroascorbic acid is fast, and the cathodic shift in the oxidation potential indicates that dehydroascorbic acid produced is adsorbing preferentially on the CNFs. This effect is observed to be strongest on the longest CNFs, most likely because they have the largest amount of reactive fiber area.

### Selectivity

**Figure 6:**
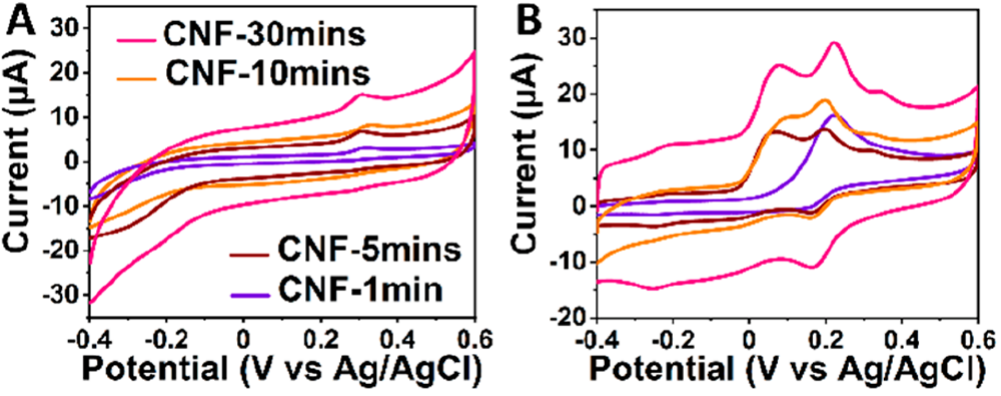
CVs recorded in (A) 50 μM UA and (B) ternary solution of 100 μM DA, 1 mM AA, and 50 μM UA in PBS for CNF electrodes grown for different durations.

The ability of the CNF electrodes with varying lengths to detect and distinguish DA in the presence of AA and UA (major interferents in the brain tissues) was studied next. CV measurements were performed in the ternary solution containing 100 μM DA, 1 mM AA, and 50 μM UA in PBS at 100 mV/s (Figure 6 and Table 4). Interestingly, CNFs grown for 5–30 min showed distinct peaks for DA, AA, and UA contrary to the CNF-1min electrodes. In comparison to the measurements done separately in the solutions of 1 mM AA, 100 μM DA, and 50 μM UA in PBS, the measurements in the ternary solution of all three components showed an anodic shift in oxidation peaks for all electrodes. These shifts of oxidation peaks of DA and AA in the anodic direction can be explained by the known chemical interactions of DA with AA in the solution.^30^ However, the oxidation potential of UA did not show notable changes as a function of CNF length indicating that the UA (or its oxidation products) is not adsorbing preferentially as an effect of changes in the CNF length (Figure 6A). The key to the selectivity in this system can be summarized as follows: (i) adsorption of DA on CNFs shifts its oxidation peak to anodic direction, while (ii) adsorption of dehydroascorbic acid shifts the oxidation peak of AA into the opposite direction (cathodic), and therefore (iii) separation between the oxidation peaks of AA and DA increase. (iv) As the effect is stronger in the longer CNF (at least in the length scales studied here), we can (v), simply by adjusting the length of the fiber, control the separation between AA and DA peaks.

**Table 4:** Electrochemistry of Uric Acid and Selectivity Trends Shown by Peak Positions of AA, DA, and UA on CNF Electrodes with Varying Dimensions

	50 μM UA		100 μM DA + 1 mM AA + 50 μM UA (mV)		
electrode	*E*_pa_ (mV)	*I*_pa_ (μA)	*E*_pa_ (AA)	*E*_pa_ (DA)	*E*_pa_ (UA)
CNF-30mins	326 ± 16	1.3 ± 0.4	52 ± 12	216 ± 11	341 ± 7
CNF-10mins	334 ± 20	1.7 ± 0.3	82 ± 9	202 ± 4	326 ± 7
CNF-5mins	345 ± 14	1.7 ± 0.1	73 ± 5	198 ± 1	326 ± 1
CNF-1min	335 ± 15	2.5 ± 0.7		220 ± 5	

### Rotating Disk Electrode Measurements

**Figure 7:**
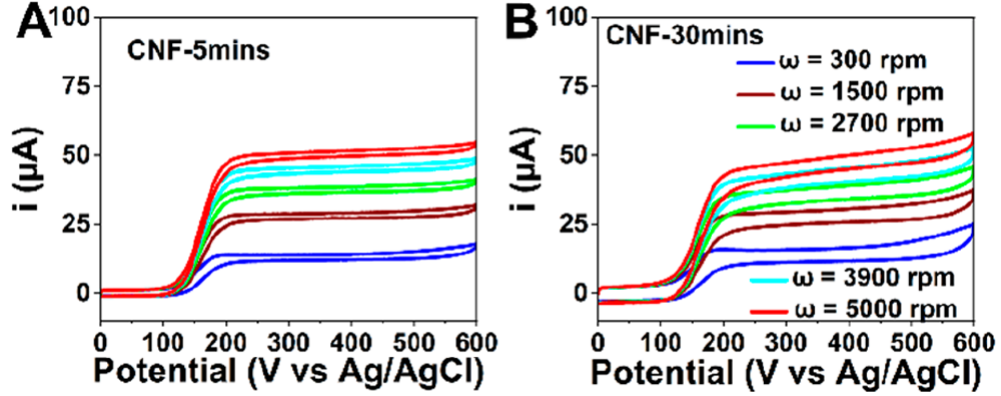
Hydrodynamic voltammograms of (A) CNF-5mins and (B) CNF-30mins recorded in 100 μM DA in PBS at 10 mV/s.

To obtain results under steady-state conditions and compare them to the results acquired from the transient analyses (CV), we utilized the RDE configuration. Hydrodynamic voltammograms for CNF-5mins and CNF-30mins electrodes are provided in Figure 7. The hydrodynamic voltammograms for CNF-30mins exhibit more hysteresis in comparison to CNF-5mins, suggesting that the effect of adsorption of DA is more enhanced on longer fibers. In addition, the changes in the slopes of the plots as a function of rotation speed in the mixed region (where both reaction kinetics and mass transfer contribute to the overall kinetics of the reaction) are less substantial in comparison to CNF-5mins. This indicates that adsorption has a strong role in the reaction at all rotation rates.

Mass transport and kinetic parameters of CNF-5mins and CNF-30mins were subsequently estimated utilizing Levich and Koutecký-Levich in 100 μM DA solution (Figure S6). The Levich plot (350 mV) for CNF-5mins increases linearly with the square root of the rotation rate and passes through the (almost) origin indicating that the reaction is governed solely by mass transport (Figure S6).^31^ The slopes of the 5 and 30 min CNFs for the Levich plots were approximately the same, indicating that under steady-state conditions the diffusion behavior is about the same (Table 5). The kinetic current estimated from the rising part of the voltammograms based on the Koutecky–Levich analysis^32^ is slightly higher for CNF-30mins in comparison to CNF-5mins. Consequently, the apparent standard heterogeneous rate constant is also showing increasing trend for the electrodes with longer CNF in comparison to shorter CNF, again indicating the importance of the fiber length in the overall reaction kinetics.

**Table 5:** Kinetic and Mass Transfer Parameters Determined from RDE Voltammograms for CNF-5mins and CNF-30mins Electrodes

electrode	*k* (cm/s)	*D* (cm^2^/s)	*i*_kin_ (mA)
CNF-5mins	0.002 ± 0.000	3.20 × 10^–7^ ± 2.35 × 10^–8^	0.02 ± 0.00
CNF-30mins	0.007 ± 0.005	3.08 × 10^–7^ ± 5.6 × 10^–8^	0.04 ± 0.03

## Conclusions

The geometry of the CNF electrodes was controlled systematically by regulating the growth parameters. Electrodes with varying CNF lengths were fabricated with similar surface chemistries, and the effect of changes in the geometry on redox reactions of DA, AA, and UA was studied. The following major observations were made: (i) Increasing the length of the fibers increased the sensitivity toward DA. (ii) The adsorption of DA on CNFs induced an anodic shift to the oxidation potential, while (iii) adsorption of dehydroascorbic acid on CNFs induced an opposite cathodic shift to the oxidation reaction of AA. (iv) Both phenomena became more important as the length of the fibers increased. This implies that (v) we can, merely by controlling the length of the CNFs, control the peak separation between AA and DA oxidation and thus the selectivity. It should also be noted that the UA oxidation peak location was not a strong function of the fiber length. Hence, this work presents a model system pointing toward the immense potential of slight modification of the geometry of the CNFs to solve some of the critical issues in the field of electroanalytical chemistry.

## Supporting Information Available

Additional details of calculation of parameters, theory of rotating disk electrode method, SEM images, atomic percentages for XPS, and other electrochemical results (PDF)
