# Supplementary material for: Modulating the Geometry of the Carbon Nanofiber Electrodes Provides Control over Dopamine Sensor Performance
Source: Anal Chem. 2023 Jan 26;95(5):2983–91. doi: 10.1021/acs.analchem.2c04843 (PMC9909731; doi:10.1021/acs.analchem.2c04843)
Supplement: Supplementary file 1 — ac2c04843_si_001.pdf [file ac2c04843_si_001.pdf]

Supporting Information

Modulating the Geometry of the Carbon Nanofiber Electrodes Provides the Control over Dopamine Sensor Performance

*Ayesha Kousar[a], Ishan Pande[a], Laura F. Pascual[a], Emilia Peltola[a b], Jani Sainio[c] and Tomi Laurila[a,d]**

[a] Department of Electrical Engineering and Automation, School of Electrical Engineering, Aalto University, PO Box 13500, 00076 Aalto, Finland

[b] Department of Mechanical and Materials Engineering, Faculty of Technology, University of Turku, Vesilinnantie 5, 20500 Turku, Finland

[c] Department of Applied Physics, School of Science, Aalto University, PO Box 15100, 00076 Aalto, Finland

[d] Department of Chemistry and Materials Science, School of Chemical Engineering, Aalto University, PO Box 16200, 00076 Aalto, Finland

*tomi.laurila@aalto.fi

Table of Contents:

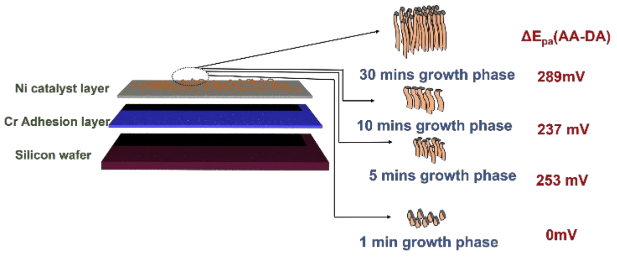

The double-layer pseudocapacitance (C_{dl}) for the electrodes was calculated from the CV recorded at different scan rates (10 mV/s-400 mV/s) by following the $\Delta i=2\times C\times v$ equation. To calculate the pseudocapacitance, differences in anodic and cathodic current from the electrical double layers were plotted against scan rates, and the slope of the plot was divided by the geometric area of the corresponding electrode. A threshold density of ±150 μA/cm² was used for measuring the potential windows. Levich plots were drawn to study the mass transport effects in the limiting current according to the following equation[1].

$$\frac{1}{i_L} = \left(\frac{1}{0.620nFD^{\frac{2}{3}}\, v^{-\frac{1}{6}}C}\right)\omega^{-1/2} \qquad \text{(S1)}$$

Where n is the number of electrons transferred during the electrochemical reaction, F is the Faraday's constant, D is the diffusion coefficient of the reactant, v is the kinematic viscosity of the electrolyte, C is the concentration of the reactant in bulk solution, and $\boldsymbol{\omega}$ is the rotational frequency. To investigate the kinetic current contributions in the case when the reaction is limited by both mass transport and sluggish kinetics, Koutecký–Levich equation (given below) is utilized. Kinetic current (i_{kin}) can be measured by extrapolating the linear combination of $1/i_L$ vs $\boldsymbol{\omega}^{-½}$ to the origin, where the intercept gives the reciprocal of kinetic current[2].

$$\frac{1}{i_L} = \frac{1}{i_K} + \left(\frac{1}{0.620nFD^{\frac{2}{3}}\, v^{-\frac{1}{6}}C}\right)\omega^{-1/2} \qquad \text{(S2)}$$

Standard rate constants for different electrodes were measured using the equation given below from the i_{kin} values obtained from Koutecký–Levich plots.

$$i_k = nFk_hC^b \qquad \text{(S3)}$$

Where K_h is the rate constant for heterogeneous electron transfer, A is the geometric area of the electrode surface and C^b is the concentration of the reactant in bulk solution.

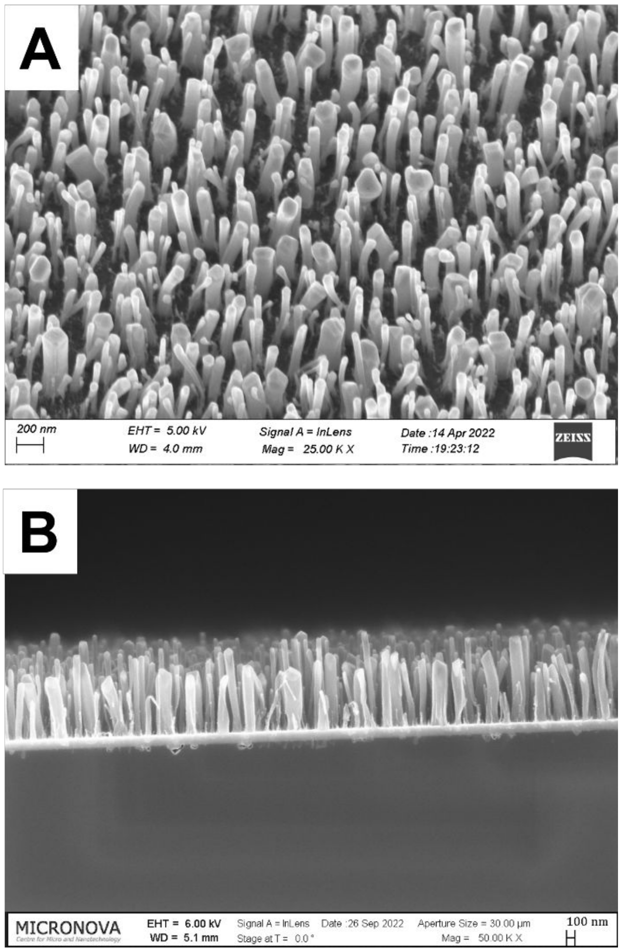

Figure S1. SEM image of CNF-10mins (A) top view by tilting the sample to 25° (B) Cross sectional view.

Table S1. Electrochemistry of $[Ru(NH_3)_6]^{3+}$ in 1M KCl on CNF electrodes with different lengths at 400mV/s *v*.

**Sample**	**ΔE_p (mV)**	**I_{pa} (µA)**	**I_{pa}/I_{pc}**	**k_0 (cm/s)**
CNF-30mins	53 ±2	27 ± 1	0.87±0.01	0.517 ± 0.000
CNF-10mins	61 ± 2	24 ± 2	0.89±0.03	0.312 ± 0.154
CNF-5mins	59.5 ± 2.5	23 ± 1	0.96±0.04	0.401 ± 0.097
CNF-1min	60.8 ± 2.0	25 ± 1	0.93±0.01	0.339 ± 0.101

Table S2. The atomic percentages (at-%) of the elements for CrNi-CNF-5 min and TiNi-CNF-30 min samples including peak fitting results. The error associated with each value is roughly ±10% of the value.

**Element**	**CNF-5mins**	**CNF-30mins**
**C of which**	**55.5**	**55.7**
sp2 C	16.7	18.3
sp3 C / sp2 C−N	17.8	18.8
C−O−C / C−OH / sp3 C−N	7.1	6.2
C=O	4.4	4.8
O−C=O	8.3	6.3
π−π*	1.2	1.3
**O of which**	**27.2**	**25.2**
O−Metal	1.2	1.4
O=C	15.9	17.0
O−C / OH−C	10.1	6.8
**N**	**8.5**	**9.2**
**Cr**	**4.2**	**3.6**
**Ni**	**4.0**	**4.1**
**Si**	**0.6**	**2.2**

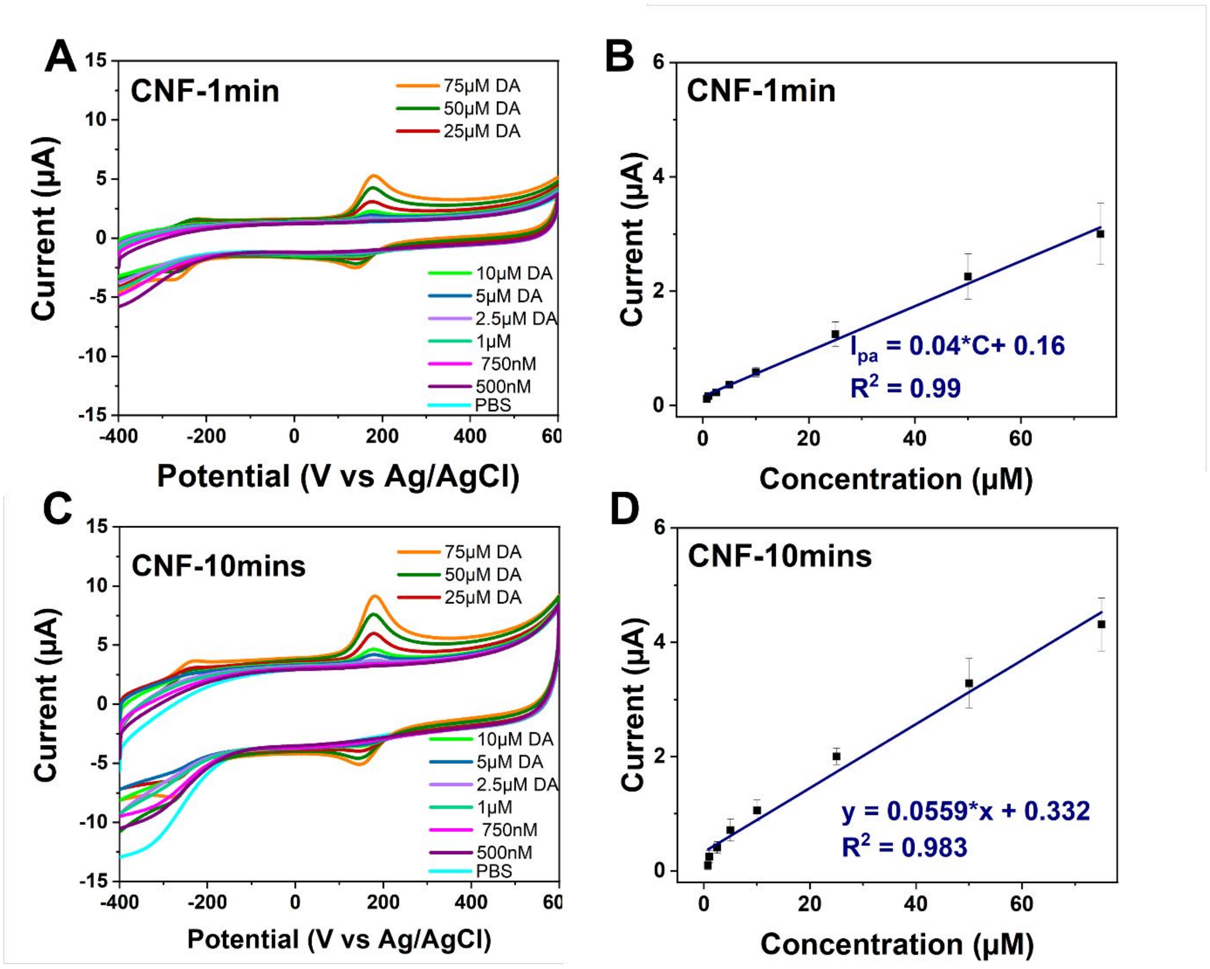

Figure S2. CVs and calibration curves of oxidation current vs concentration for (A and C) CNF-10 mins and (B and D) CNF-5mins electrodes.

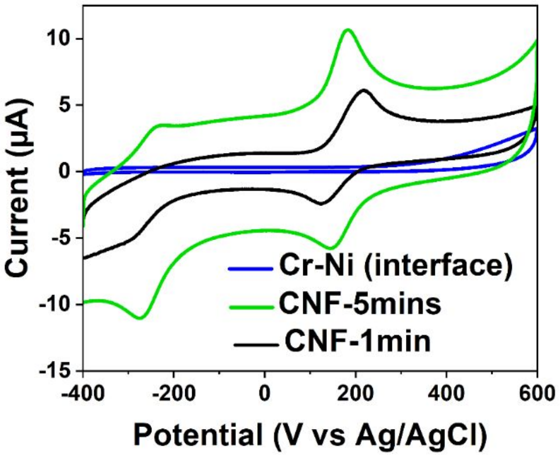

Figure S3: Comparison of metal interface with the CNF electrodes

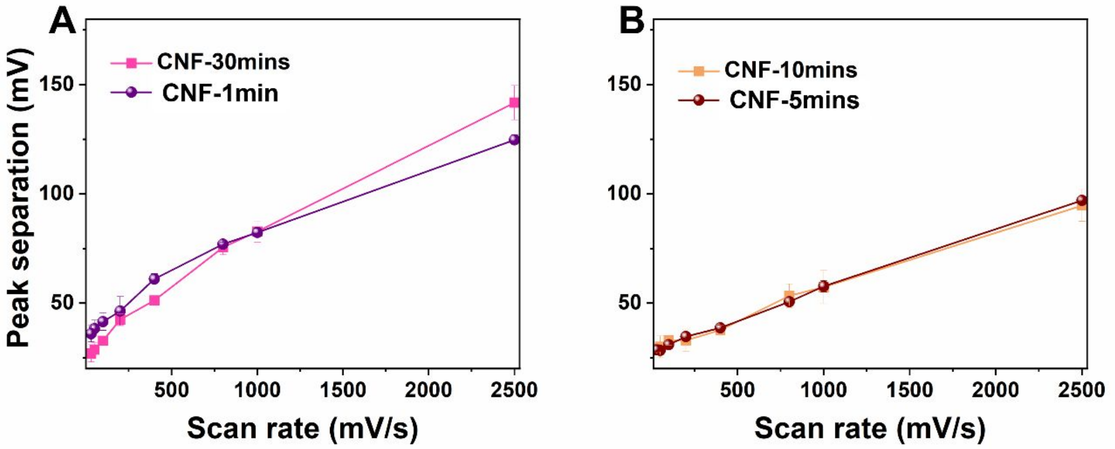

Figure S4: Plots of peak separation (ΔE_p) as a function of scan rate at 100 μM DA in PBS for (A) CNF-30mins and CNF-1min and (B) CNF-10mins and CNF-5mins electrodes.

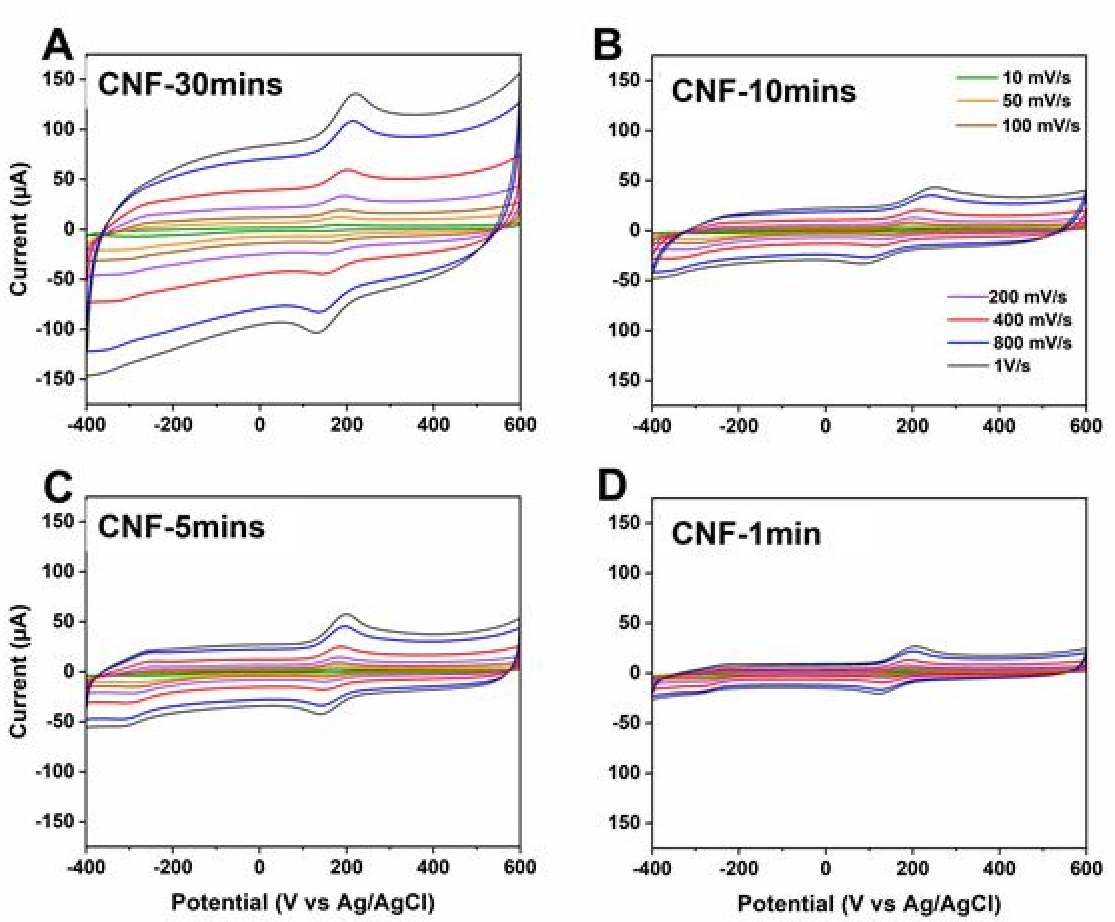

Figure S5. CV response of electrodes (A) CNF-30mins (B) CNF-10mins (C) CNF-5mins (D) CNF-1min at various scan rates (10 mV/s-1V/s) in 100μM DA concentration in PBS electrolyte.

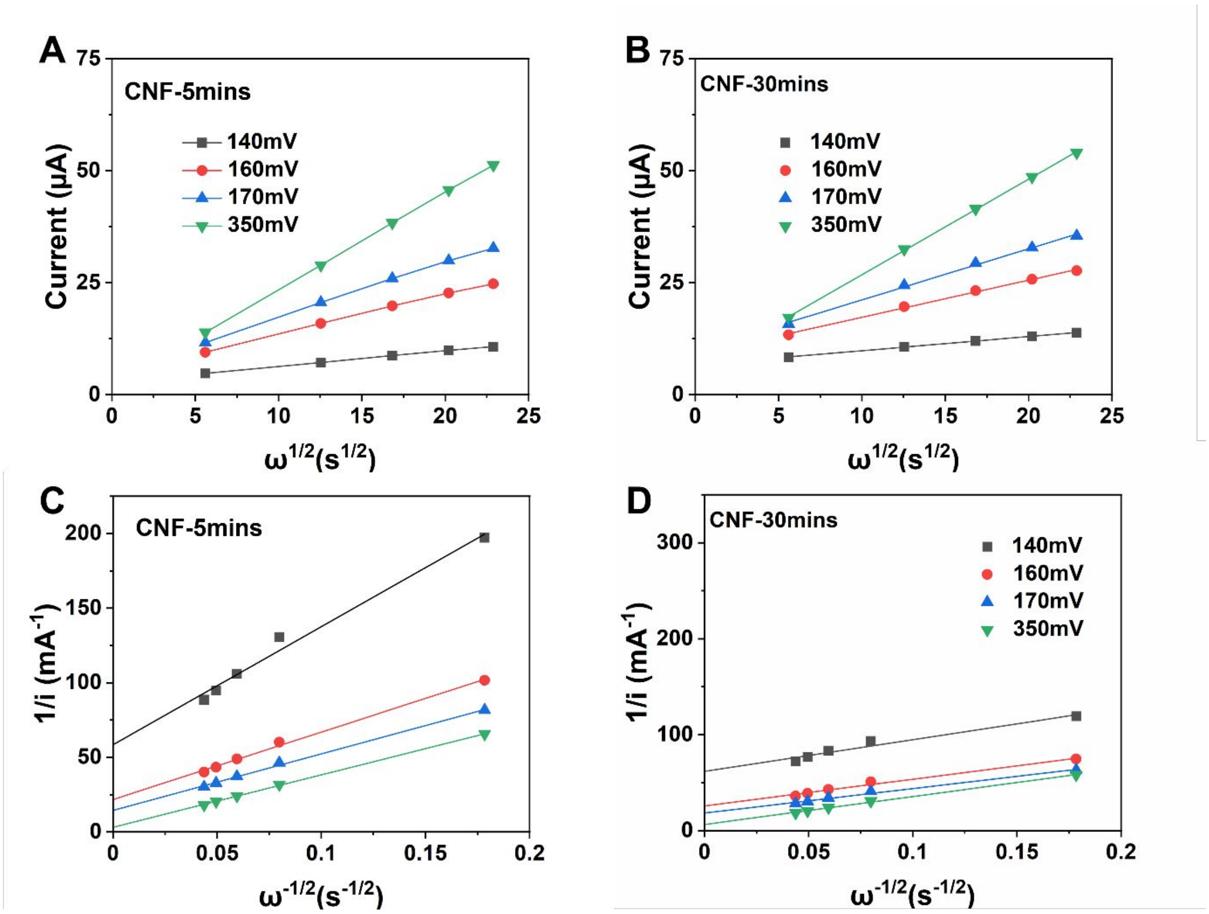

Figure S6. Levich (A and B) and Koutecký–Levich plots (C and D) for CNF-1 min and CNF-30mins electrodes

References

(1) Bard, A. J.; Faulkner, L. R. ELECTROCHEMICAL METHODS Fundamentals and Applications. *Surf. Technol.* **1983**, *20* (1), 91–92.

(2) Treimer, S.; Tang, A.; Johnson, D. C. A Consideration of the Application of Kouteck{\`y}-Levich Plots in the Diagnoses of Charge-Transfer Mechanisms at Rotated Disk Electrodes. *Electroanalysis* **2002**, *14* (3), 165–171.
